# Umut Şahin: SUMOylation in health and disease

**DOI:** 10.26508/lsa.202201534

**Published:** 2022-06-03

**Authors:** Umut Şahin

**Affiliations:** Associate Professor of Molecular Biology and Genetics, Center for Life Sciences and Technologies, Boğaziçi University, Istanbul, Turkey

## Abstract

Umut Şahin is an Associate Professor in the Department of Molecular Biology and Genetics at Boğaziçi University’s Center for Life Sciences and Technologies. We asked him about his recent paper published in *Life Science Alliance* (*LSA*) and his experience in science thus far. He explains how his team built upon their initial in vivo data by further complementing it with in vitro work, and emphasizes the relevance of their study to virology, immunology, as well as to the SUMO community. He shares the long term goals and vision of his laboratory, and explains how past and current connections shape his research interests and fuel his passion for science.

## Abstract (precis)

Umut Şahin is an Associate Professor in the Department of Molecular Biology and Genetics at Boğaziçi University’s Center for Life Sciences and Technologies. We asked him about his recent paper published in *Life Science Alliance* (*LSA*) and his experience in science thus far.

**Figure 1:**
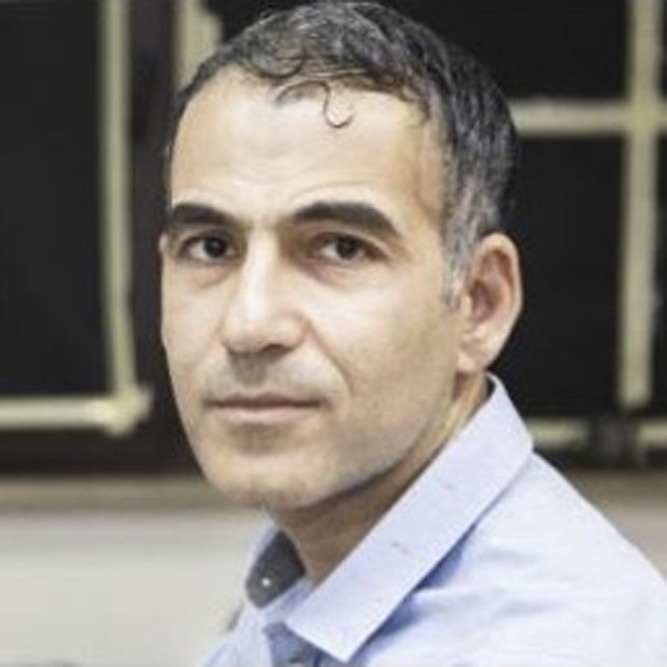
Umut Şahin

## How would you explain the main findings of your paper (1), and how did it come about?

My lab is primarily focused on investigating the roles of SUMOylation, a key post-translational modification which has attracted immense attention over the past 2 decades as a crucial mechanism for development and stress response, in major cellular processes, particularly in the context of pathology. Initially, we were interested in studying the potential changes in proteome-wide sumoylation in vivo among people suffering from chronic inflammation. Numerous diseases, including cancer, arthritis, neurodegeneration, multiple sclerosis, metabolic syndrome, diabetes and infections, are associated with chronic inflammation that can cause severe damage to the body. Altered (possibly increased) sumoylation may serve as a target process that can potentially be manipulated with therapeutic benefits.

Because untreated HIV disease is notoriously associated with chronic inflammation, it was included on our list of conditions that we initially aimed to study with respect to alterations in in vivo sumoylation. While studying the blood of HIV-infected individuals, more specifically, their white blood cells, we consistently observed a proteome-wide reduction in the sumoylation activity. This disruption in sumoylation in vivo was so persuasive and compelling that we have decided to focus on HIV infection for the time being and explore this phenomenon in further detail.

In retrospect, the story of this paper has evolved in a rather untraditional manner in that some unexpected yet very strong findings in an in vivo setting prompted us to conduct further in vitro research, and not vice versa. Exceptions aside, often in biomedical research, in vitro work precedes in vivo studies, meaning that we usually require sufficient in vitro evidence to justify long-term investment in animal or patient-based studies. Indeed, because of the elevated levels of inflammation and the persistent stress burden on the immune system in HIV disease, we initially expected to observe a subtle upregulation of sumoylation in vivo, or system-wide changes that would perhaps only be detected in proteomic analyses. Nevertheless, the sharp decrease we see in the abundance of global SUMO conjugates in the leukocytes was also perfectly consistent with the growing literature on how pathogens attack sumoylation to disarm host immune responses. Therefore, to explore this further, we decided to carry out elaborate ex vivo studies, and eventually showed that HIV-1 expression in cell culture was sufficient to cause a sharp and specific loss of a subunit of the SUMO E1 enzyme, wiping out cell-wide sumoylation as a consequence. Eventually, when we wrote the paper, we chose to introduce the in vitro results first, which were then supported by the data coming from the patients, because it simply made more sense in that order and allowed everything to come together as one coherent story. The pandemic has really focused many scientists’ attention on pathogens’ influences on human life and on society. Humankind has been living with viruses for millennia, constantly co-evolving. HIV infection is an ongoing pandemic and still a major public health issue. In this paper, we have unraveled a new and previously unexplored layer of complexity by which this virus targets our immune system.

Both my PhD and postdoc advisors (Carl Blobel in New York and Hugues de Thé in Paris, respectively) were MD, PhDs. I believe that training with them and observing how they approached basic scientific problems from a medical perspective also provided me with a certain insight to address some of the existing gaps in biomedical knowledge and therapies. In that respect, with this particular study on HIV, I was very glad to have the opportunity to collaborate with some of the best medical doctors in Turkey.

## What was the decision process in choosing where to publish?

As scientists, we strive to publish in eminent journals, which ensures visibility. Accomplishing this also validates the relevance of our work straight away, even before the citations start kicking in. Part of that validation process involves robust peer-review. If you get constructive and positive feedback from the experts in your field who can also point at the weaknesses of your original submission in a balanced manner, if you get a feeling that they have really invested their time in reading your manuscript and going over your data, and that they are willing to cooperate with you and with the journal editors to further improve your paper before accepting and presenting it to the field, then not only do you feel that your work raised interest and attracted attention, but also that you are in the right place to publish.

This is exactly how I can describe my experience with *LSA* regarding the two other papers that I had previously published in this journal prior to our story on HIV. I was first introduced to *LSA* a few years ago during one of the annual EMBO YIP meetings in Heidelberg, immediately following the launch of this journal. I had the opportunity to hear first-hand about its mission and vision from the editors at EMBO Press, and even learned a bit about the story behind its launch from Tilmann Kiessling, the head of EMBO Communications, when he visited us in Istanbul a few years ago. With the support of three prominent and reputable press houses and a stellar list of advisory editorial board members, I immediately realized that achieving publication at *LSA* would ensure visibility.

The peer review process of my first two publications at *LSA* was indicative of high editorial and referee standards, almost comparable to my past experiences with the Journal of Cell Biology (published by one of the alliance partners, the Rockefeller University Press) where I have two first author papers. In the meantime, the editorial process was so smooth, rapid and transparent, and the editors were so understanding, communicative and cooperative that I did not hesitate to send our most recent story also to *LSA* as well. In brief, a few things factored in my decision-making process in submitting our work to *LSA*: prestige, visibility, high scientific standards and robust peer review, smooth and friendly editorial process, transparency, and open-accessibility. And of course, importantly, so far I have really enjoyed reading many of the publications that came out in this journal.

## How do you think publishing in an open access journal like Life Science Alliance has impacted the visibility of your findings?

Especially during this pandemic, I think most of us have acknowledged the importance of promptly disseminating the scientific knowledge and progress, not only to our colleagues in our own field of study, but also to the scientists from other disciplines, as well as to the public. I have always had the chance to work at prominent institutions with reasonable access to many scientific and medical journals. But even then, I have been frustrated on so many occasions for not being able to download a specific article with a very appealing title and abstract, either due to lack of subscription or I was simply off campus and had no access to the journal. When these circumstances present, either I have to remember to chase after that article (and this task adds to my already crowded to-do list) or sometimes I simply give up and move on. Therefore, from my own experience and perspective, I feel that publishing in an open access journal impacts the visibility of our work in a positive way, and allows our findings and discoveries to reach more people, including researchers that may be working in low- or mid-income countries where their host institutions may not warrant subscriptions to many scientific journals. In addition, while academic institutions usually offer broad access to scientific journals, many hospitals and pharmaceutical companies do not. Because my work also interests medical doctors who may be working in hospitals, as well as drug discovery teams in the pharmaceutical industry, publishing in an open access journal like *LSA* allows me to reach out to them.

## What advice do you have for other researchers on maximizing the dissemination of their work?

Publishing in a visible, trustworthy and reputable journal definitely helps, and I have learned that these do not necessarily align with the impact factor. Therefore, my first advice would be to pick a journal where your work really fits in: finding the most suitable journal for your work is an important but often underestimated task, and this will not only affect the initial editorial evaluation process, but also eventually determine who will see and read your paper.

Next, make sure that the journal features the abovementioned qualities: visible, trustworthy and reputable (once again, this last one is not necessarily reflected in the impact factor or determined by how old the journal is). Finally, one should keep in mind that publishing in an open access journal will no doubt enhance visibility.

Although publication is an important part of the research process, dissemination of our work also involves conferences, meetings, symposia and seminars. Another piece of advice would be to attend such events as frequently as possible and make an effort to present your work. If possible, also take part in the organization process. These events present great opportunities for networking, reaching out, making yourself and your work visible, and getting valuable feedback from your colleagues and peers from all over the world.

Finally, I have recently discovered that many scientists are also very active on social media, especially on Twitter, which, I myself have also started using. This really helped me make connections with scientists from all over the world, some within my own field of research and others from many diverse fields, and allowed me to follow their scientific progress and to observe their academic (or non-academic) journeys. On social media, scientists also share their problems and frustrations with which I can easily empathize. Particularly during the pandemic, using social media for science (Twitter, specifically) helped me feel less isolated and better-integrated with the rest of the world, and allowed me to stay up-to-date on the latest scientific developments and policy making.

## What questions is your lab currently trying to answer?

The long term goal of my research is to understand the biology of SUMO proteins, to dissect their functions in health and pathology, and to explore their potential for targeted therapies. We use multidisciplinary approaches such as cell biology, biochemistry, biophysics, high resolution imaging, proteomics, as well as transgenic animal models of disease. We also work very closely with medical doctors.

Many key proteins are now emerging as prime sumoylation targets, and our core scientific interest is to define how sumoylation regulates their function. For example, we have recently reported in *LSA* our discovery of Cas9 sumoylation (2), the first post-translational modification to be ever described on this very important enzyme. Even though the CRISPR/Cas9 system is a popular genome editing platform with many future prospects for therapeutics, surprisingly, little is known about how this system behaves in human cells. We showed that Cas9 modification by the human SUMO peptides modulates the stability of this enzyme, as well as its binding ability to target DNA sequences.

A major question that my lab is currently researching is whether this modification actually affects the DNA cleavage and genome editing activities of Cas9, and if so, how. We are also investigating the impact of sumoylation on Cas9’s off–target activities, which, from a clinical perspective, are of great concern for the use of CRISPR in humans to cure disease.

In parallel, we are trying to understand the evolutionary significance of Cas9, a bacterial protein, undergoing a eukaryotic modification (SUMO). Is this meaningful from a host–pathogen interactions standpoint? Several viral and bacterial proteins are known to be sumoylated following infection of the host, and this may have positive or negative outcomes for the pathogen, depending on the context. *Streptococcus pyogenes*, the original source of the most commonly used Cas9 variant across the globe, is actually a common pathogen. I am very intrigued by the possibility that Cas9, during infection, may actually function in an obscure, yet-to-be discovered manner that might actually justify its sumoylation by the host eukaryote. We are currently trying to address this question.

I am also highly interested in exploring the potential of sumoylation for the development of next generation targeted therapies. Lately, we have focused primarily on ALS (amyotrophic lateral sclerosis), a neurodegenerative disease with dismal prognosis, and mainly studied the functions of sumoylation in the pathogenesis of this disease. Specifically, we strive to better understand the molecular and cellular basis of neurodegeneration in ALS patients, especially in those with previously unexplored mutations. For instance, we are investigating the liquid–liquid phase separation behavior of ALS-linked proteins, as well as their tendency to escape from cellular quality control, and trying to understand how these processes may be affected by (dysregulated) sumoylation. I have good reason to believe that the latter may serve as a target process that may be manipulated pharmacologically for therapeutic purposes. To study this, we are also using novel animal models of ALS, and some preclinical studies are already underway. Hopefully, we have a big story coming up on this.

On other fronts, we are also keen on studying the roles of sumoylation in metabolic regulation, as well as in cancer processes.

## What motivated you to pursue a career in science, and what have been the most interesting moments on the path that led you to where you are now?

I come from a family of judges, attorneys and prosecutors. There were no scientists, physicians or teachers in my family that would otherwise inspire me to do science. My parents encouraged me to go to law school, but I had no spark of interest or excitement for that subject. I grew up watching many of the classic sci-fi movies and TV shows of the 80s and 90s, and I have always been fascinated by their various depictions of humanity’s future, the role of science, technology and innovation in our society, humankind’s vast understanding of the universe and its command on the laws that govern it. Reading popular science and about our nature had become a habit pattern for me, and pursuing a career in science only felt like second nature. Eventually, biomedical and health sciences have become my passion, but I am also generally interested in other scientific disciplines as well, physics in particular. For example, I am really fascinated by experimental high energy physics and cosmology, and try to keep myself up-to-date on our endeavor, as humankind, to explore fundamental questions about the nature of our universe.

There were indeed defining moments along my career path that led me to where I am today. An interesting one worth mentioning is when I faced a crossroads to choose a lab to pursue my PhD studies. Back in 2001, I was almost 1.5 years into my graduate studies at Brown University’s Molecular and Cellular Biology and Biochemistry Program. I was working, happily, in a protein biochemistry lab where I thought I would complete my PhD thesis. One morning, I woke up to the news that my prospective thesis advisor was not granted tenure. I remember feeling really frustrated and anxious, and having already completed three lab rotations, I was not sure which lab to transfer to start things over. Then, following discussions with our graduate program head and the dean, I filed an application (which was then promptly granted) for a special program which allowed outstanding graduate students enrolled in a PhD program in one of a dozen participating institutions to study at one of the other participating universities, which included all eight Ivy League schools, MIT, Stanford and UC Berkeley. Suddenly, I was presented with an incredible opportunity: I could pick from a list of hundreds of labs situated at these excellent host institutions and pursue my PhD career there. I was primarily interested in protein biochemistry and folding, post-translational modifications, proteolysis and degradation.

I had discussions with several PIs over the course of the next few weeks, and finally ended up joining Carl Blobel’s lab at Cornell Medical School and Sloan-Kettering Institute in New York, which has been a landmark point in my career. In retrospect, this was an excellent choice and I still feel very lucky that Carl had an opening in his lab at that very moment. I greatly enjoyed my time in his lab, all the while getting an excellent training. Together, we wrote a now-classic Journal of Cell Biology paper on the ectodomain shedding of the EGFR ligands, which, after 17 years of its publication, still gets cited once every few days (3). Along the years, the tri-institutional campus that hosts MSKCC, Cornell Medical School and Rockefeller University presented me with quite a few opportunities to meet many great scientists, some with really colorful characters.

Aside from some brief periods of frustration, in general, I feel extremely lucky to have always worked with amazing scientists, who were also intellects, great supervisors and friends at the same time. Another breakthrough moment on my career path was joining Hugues de Thé’s lab in Paris for my post-doctoral studies. Working with Hugues, and also with Valérie Lallemand-Breitenbach, ultimately shaped and defined my interest in sumoylation as a post-translational modification, from a standpoint of both basic biology and translational medicine.

## Tell us something interesting about yourself that would not be on your CV

I have great enthusiasm for science communication, innovative dissemination of research and scientific progress. Several years back, while I was still a postdoc in Hugues de Thé’s lab, I actually applied for a scientific editor position at a competitive cancer journal. My application was shortlisted, which was followed by a rigorous selection process during which I successfully completed the assignments I was given, went through phone interviews, and finally got invited to their headquarters for a final in-person person interview. When this job and the new career path started to become a reality for me, which I would have no doubt enjoyed, it also dawned on me that I actually wanted an active career in research, so I ended up declining this final interview offer. Today, as an academician, I still try to contribute as much as I can to the process, for instance, by writing reviews for the field, in particular with a historical perspective, or by actively reviewing manuscripts.

Also, one day, I would like to get my master of arts degree in Asian Studies, if time allows. I have great interest in Asian and Southeast Asian cultures, history, politics, languages, and of course, cuisine. Several years ago, I took up the challenge of learning Mandarin Chinese and it has been an intense and very interesting cultural and philosophical journey for me. I am interested in the philosophy of language as a discipline, and fascinated by the interplay between the mind, thought, language and communication.
